# The Effect of Telomerase Inhibition on NK Cell Activity in Acute Myeloid Leukemia

**DOI:** 10.34172/apb.2023.018

**Published:** 2021-10-10

**Authors:** Khadijeh Dizaji Asl, Ali Rafat, Ali Akbar Movassaghpour, Hojjatollah Nozad Charoudeh, Hamid Tayefi Nasrabadi

**Affiliations:** ^1^Student Research Committee, Tabriz University of Medical Sciences, Tabriz, Iran.; ^2^Department of Anatomical Sciences, Faculty of Medicine, Tabriz University of Medical Sciences, Tabriz, Iran.; ^3^Hematology and Oncology Research Center, Tabriz University of Medical Sciences, Tabriz, Iran.; ^4^Stem Cell Research Centre, Tabriz University of Medical Sciences, Tabriz, Iran.

**Keywords:** Natural killer cells, Telomerase, Telomerase inhibitor, Acute myeloid leukemia

## Abstract

***Purpose:*** Acute myeloid leukemia (AML) is known to be an invasive and highly lethal hematological malignancy in adults and children. Resistance to the present treatments, including radiotherapy and chemotherapy with their side effects and telomere length shortening are the main cause of the mortality in AML patients. Telomeres sequence which are located at the end of eukaryotic chromosome play pivotal role in genomic stability. Recent studies have shown that apoptosis process is blocked in AML patient by the excessive telomerase activity in cancerous blasts. Therefore, the find of effective ways to prevent disease progression has been considered by the researchers. Natural killer (NK) cells as granular effector cells play a critical role in elimination of abnormal and tumor cells. Given that the cytotoxic function of NK cells is disrupted in the AML patients, we investigated the effect of telomerase inhibitors on NK cell differentiation.

***Methods:*** To evaluate telomerase inhibition on NK cell differentiation, the expression of CD105, CD56, CD57, and KIRs was evaluated in CD34^+^ derived NK cells after incubation of them with BIBR1532.

***Results:*** The results showed that the expression of CD105, CD56, CD57, and KIRs receptors reduces after telomerase inhibition. According to these findings, BIBR1532 affected the final differentiation of NK cells.

***Conclusion:*** The results revealed that telomerase inhibitor drugs suppress cancer cell progression in a NK cells-independent process.

## Introduction

 Acute myeloid leukemia (AML) is a highly lethal hematological malignancy in adults and children. It is characterized by the abnormal proliferation and accumulation of leukemia stem cells (LSCs) in the bone marrow and peripheral blood.^1^ LSCs with CD34⁺ and CD38^-^ phenotype activate the cancer genes by increasing the telomerase activity and are the main cause of chemotherapy resistance in AML patients.^2^ Telomeres sequences (TTAGGG) are located at the end of chromosome.^3^ Telomere length shortening occurs with age and cell division in normal somatic cells.^4^ Although, telomere sequences are maintained by telomerase enzyme using specific RNA template,^5^ the excessive enzyme activity was reported in most transformed malignant cells.^6^ In this line, the faster disease progression and poor response to chemotherapy are seen in patients with high telomerase activity.^7^ Today, telomerase inhibitor drugs such as BIBR1532 was preferred by many researchers as an effective therapeutic options in cancer therapy. BIBR1532 is non-nucleoside pharmacological inhibitor and induce P53-dependent apoptosis in leukemic stem cells^8,9^ with no significant effect on normal cells. According to the previous results, BIBR1532 inhibits cancer cells progression in a dose-dependent manner.^10^ Natural killer (NK) cells as granular effector cells have the main role in elimination of transformed and tumor cells.^11^ They are defined with CD56^+^CD3^-^ phenotype^12^ and arise from CD34 positive hematopoietic stem cells in the bone marrow.^13^ Overall, NK cells function is controlled by the transmitted signals from the activating and inhibitory receptors. There is some evidence that NK cell’s cytotoxic function is disrupted in AML patients, and tumor cells escape from killing by NK cells with the secretion of soluble factors and the shedding of activating ligands.^14^ Hypoxia in tumor microenvironment also lead to the disarming and production of inactive NK cells.^15^ Due to the high telomerase activity in hematological malignancies, employing appropriate strategy to produce fully functional NK cells have been attention by researchers. In this study, we evaluated the effect of telomerase inhibitors on NK cell activity. Our results revealed that the expression of CD105, CD56, CD57, and KIR receptors decreases after telomerase inhibition. According to these finding, BIBR1532 as a telomerase inhibitor, affected the final differentiation of NK cells.

## Materials and Methods

###  CD34^+^ and CD34^-^ cells enrichment

 AML cancer cell line, namely KG1-a, was purchased from the National Cell Bank of Iran (Pasteur Institute, Tehran, Iran) and were cultured in RPMI 1640 medium plus 10% fetal bovine serum (FBS) and 1% penicillin/streptomycin at 37°C. CD34 positive and negative cells in the KG1-a cancer cell line were separated using MACS cell-separation system and anti-CD34 microbeads, according to the manufacturer’s instructions. Briefly, the cells were re-suspended in 300 μL phosphate-buffered saline (PBS) containing 0.5% BSA and incubated with 100 μL blocking antibody and anti CD34 microbeads (Miltenyi Biotec, Berlin, Germany) at 4°C. Following 30 minutes incubation, the stained cells were passed through the LS column, and then CD34 positive cells were collected with flashing in a 15 mL tube. For obtaining a high purity of the CD34 positive cells, this protocol repeats twice. To determine the viability of the cells, the enriched cells were stained with 4% trypan blue. 5 μL of cell solution was mixed with 95 μL of trypan blue. The number of cells present in the 4 squares of the Neubauer chamber was counted under an invert microscope. The percentage of CD34 positive cells was assessed by flow cytometry before and after enrichment.

###  Culture condition and cytokines

 CD34^+^ and CD34^-^ cells were seeded in 96-well plates at a concentration of 5× 10^5^ cells per well in 200 μL of RPMI (Gibco) containing 10% FBS (Gibco), 1% penicillin/streptomycin (Sigma, St Louis, MO, US), SCF, FLT3, IL-2, and IL-15 (eBioscience Company) cytokines. The final concentration of all cytokines was 50 ng/mL. The cells were incubated at 37°C and 5% CO2 for 14 days. Every week, one half of the culture medium was removed and replaced with the fresh medium (medium+ IL-2+IL-15). On day 14, 115.95 µM BIBR1532 were added to cells and NK cell differentiation was evaluated by flow cytometry.

###  Antibodies and flow cytometry analysis

 The used antibodies include: CD34 (FITC, clone 8G12, BD Bioscience) (5 μL/1× 10^6^ cells), CD3 (PE/CY5, UCHT1, BioLegend) (2 μL/1× 10^6^ cells), CD56 (FITC, clone 3G8, BD Bioscience) (2 μL/1× 10^6^ cells), CD57 (FITC) (4 μL/1× 10^6^ cells) and CD105 (FITC) (4 μL/1× 10^6^ cells). Flow cytometry was performed on day 15. Briefly, the collected cells were centrifuged at 300 g and washed twice with cold PBS plus 5% FBS (staining buffer). Then the cells spun down and were re-suspended in 50 μL of staining buffer. After adding the appropriate volume of antibodies, the cells were incubated in 4°C in the darkroom. Following 20-30 minutes incubation, the stained cells were washed with 1ml of staining buffer to remove unbounded antibodies. Finally, the cells were re-suspended in 500 μL of staining buffer and were evaluated by flow cytometry. 7-Amino-actinomycin D (7ADD, (5 μL/1 × 10^6^ ells, BD Bioscience) were used for excluding dead cells. Approximately, 10 000 to 30000 events were tracked for each samples by using BD caliber (BD eBioscience) and data were analyses by FlowJo (7.6.1) software.

###  Statistical analysis

 The software GraphPad Prism version 6.01 was used for analyzing the results. Values were expressed as the Means ± SD by triplicate independently experiments. One-way and two-way analysis of variance (ANOVA) followed by Tukey’s multiple comparisons test and Bonferroni’s multiple comparisons test were applied to determine the significant difference among groups at *P *< 0.05.

## Results and Discussion

###  Higher expression of CD34 was recorded on KG1-a cancer cell lines.

**Figure 1:**
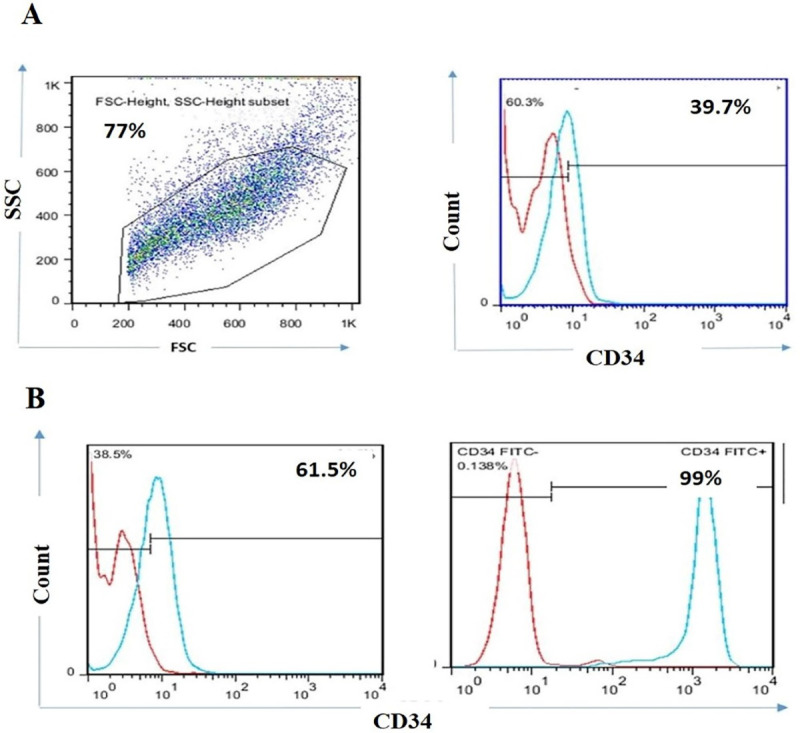
KG1-a cancer cell lines efficiently expressed CD34 positive cells. FACS plots of CD34 positive expression in KG1-a cancer cell lines before MACS (A). Representative flow cytometry profile of CD34 positive cells in one and double-positive enrichment (B). Data are representative of 3 independent experiments with 5 samples per experiment.

 At first, the expression rate of CD34 were evaluated in expanded KG1-a cancer cell lines before MACS. As shown in Figure 1A, 39.7% of KG1-a cells were CD34 positive cells. Then, CD34 positive cells were sorted from KG1-a cell line by MACS. Double enrichment of CD34 positive cells was utilized to harvest cells with high purity. The results revealed that the percentage of CD34 positive cells increased from 61.5% to 99% in the double enrichment process.

###  Lower expression of CD56/KIR and CD57 were observed in CD34^+^ derived NK cells following incubation with BIBR1532

 To determine the effect of telomerase inhibition on NK cells differentiation, we cultured CD34 positive and negative cells with IL-2, IL15, SCF, and FLT3 cytokines combination for 14 days.

**Figure 2:**
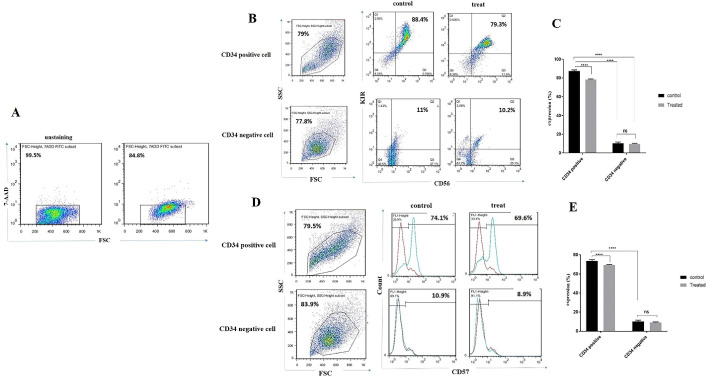
The expression of CD56/ KIR and CD57 receptors was decreased in CD34^+^ derived NK cells following the incubation of them with BIBR1532. Representative flow cytometry profile and Mean (SD) proportion of CD56 and KIR expression on NK cells (A and B). FACS profile and Mean (SD) proportion of CD57 expression on NK cells (C, D, and E). Data are representative of 3 independent experiments with 5 samples per experiment. The values are illustrated in mean ± SD. ****P< 0.0001.

 On day 14, 115.95 µM BIBR1532 was added to cultured cells and the expression of the CD56/KIRand CD57 was evaluated by flow cytometry after 24 hours. According to the 7-AAD results, the viability of differentiated NK cells was around 84% (Figure 2A). As shown in Figure 2B CD56/KIR expression was 88.4% and 79.3 in the control and treatment groups, respectively. These indicated that CD56/KIR expression is reduced in CD34 positive derived NK cells in the presence of BIBR1532 in the treatment group, however, MFI is reduced. However, no significant change was observed in CD34 negative derived NK cell with BIBR1532 (Figure 2B). For further investigation, we also evaluated the expression of CD57 in CD34 positive and negative derived NK cells. The results indicated that CD57 expression decreased from75% to 69% CD34 positive derived NK cells which were supplemented with BIBR1532 (Figure 2C and 2D). These changes were not significant between the control and treatment groups in terms of CD34 negative cells.

###  Lower expression of CD105 was recorded in CD34^+^ derived NK cells following incubation with BIBR1532

**Figure 3:**
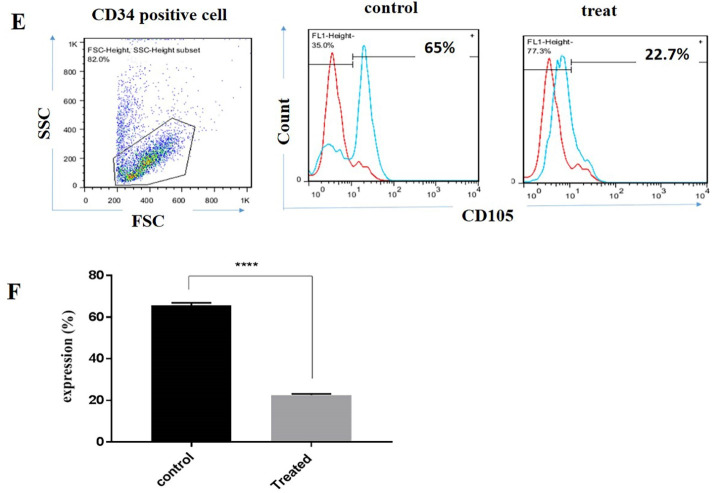
The expression of CD105 was decreased in CD34 positive derived NK cells following the incubation of them with BIBR1532. Flow cytometry profile of CD105 expression on CD34 positive derived NK cells (E). The mean ± SD percentage of CD105 in differentiated NK cells (F). Data are representative of 3 independent experiments with 5 samples per experiment. The values are illustrated in mean ± SD **** *P* < 0.0001.

 We also evaluated the effect of telomerase inhibition on CD105 expression in CD34^+^ derived NK cells. Our results indicated that, the expression of CD105 decreased from 65% to 22% when compared with the control group (Figure 3E and 3F). Collectively, our results indicated that telomerase inhibition affected the CD34^+^ derived NK cells differentiation through the down regulation of functional receptors (Figure 3).

## Discussion

 The telomeres with the TTAGGG sequence have a pivotal role in genome stability. Due to the absence of telomerase in normal somatic cells, the telomere length becomes progressively shortened with age and cell division.^16^ Telomerase enzyme with two components, including protein (hTERT) and RNA template (hTERC), prevents telomere length shortening by adding nucleotides during cell division.^17^ According to the previous studies, chromosome instability with high telomerase activity are the main cause of disease progression and poor response to chemotherapy in patients with hematological malignancies.^18^ Lymphocytes display a similar age-associated trend of telomere shortening with age.^19^ NK cells as large granular lymphocyte, have a main role in the elimination of the transformed cells. NK cells’ cytotoxic function is disrupted in tumor sites with the downregulation of activating receptors.^20^ Therefore, the finding of the best approach to maximize NK cell anti-leukemia potential may be useful in experimental or clinical applications. Although the previous studies have shown that telomerase inhibitors induce apoptosis in damaged cells and restricted tumor progression^18^ however, the relationship between telomerase inhibition and NK cell activity has not been systematically addressed. BIBR1532 suppresses the telomerase activity through the survivin-mediated c-Myc and hTERT transcript inhibition. Finally, upregulation of Bax/Bcl-2 and P53 induced apoptosis in abnormal cells.^21^ In this study, we evaluated the effect of BIBR1532 on NK cell activity for the first time. Our results showed that telomerase inhibition by BIBR1532 reduces the expression of NK cell functional receptors and NK cell activity is decreased with telomerase inhibition. In consistence with our study, Kaszubowska L, et all showed that the aging affected the functional activity and CD57 expression in NK cells.^22^ Other researchers reported that, there is a diverse relationship between the telomerase activity in NK cells with ageing.^23^ Collectively, we conclude that BIBR1532 as a telomerase inhibitor is a double-edged sword. Although BIBR1532 stimulates apoptosis in cancer cells, but it has a negative effect on the NK cell activity and induce aging in NK cells. Of course, it is reasonable to imagine that the disease severity, age, and gender among patients also have an effect on these results, which should be examined in future studies. These finding might be used as a novel method in AML patient treatment for researchers.

## Conclusion

 In summary, our results indicated that the expression of CD105, CD56, CD57, and KIRs receptors reduces after telomerase inhibition with BIBR1532. It is conceivable that BIBR1532 affected the final differentiation of NK cells with down-regulation of functional receptors. Therefore, telomerase inhibitor drugs suppress cancer cell progression in a NK cells-independent process.

## Acknowledgments

 The research protocol was approved and supported by Student Research Committee, Tabriz University of Medical Sciences** (**Grant number: 61429)

## Author Contributions

**Conceptualization: **Khadijeh Dizaji Asl.

**Data curation: **Khadijeh Dizaji Asl, Ali Rafat.

**Funding acquisition: **Ali Rafat, Khadijeh Dizaji Asl.

**Investigation: **Khadijeh Dizaji Asl, Ali Akbar Movassaghpour.

**Project administration: **Ali Akbar Movassaghpour, Ali Rafat.

**Resources: **Khadijeh Dizaji Asl.

**Supervision: **Khadijeh Dizaji Asl.

**Visualization: **Hojjatollah Nozad Charoudeh, Hamid Tayefi Nasrabadi.

**Writing – original draft: **Khadijeh Dizaji Asl.

## Ethical Issues

 This article does not contain any studies with human participants or animals performed by any of the authors (Ethical code: IR.TBZMED.REC.1397.970).

## Conflict of Interest

 All the authors declare that they have no conflict of interest
